# All framing effects are not created equal: Low convergent validity between two classic measurements of framing

**DOI:** 10.1038/srep30071

**Published:** 2016-07-20

**Authors:** Shanshan Zhen, Rongjun Yu

**Affiliations:** 1 School of Psychology, Center for Studies of Psychological Application and Key Laboratory of Mental Health and Cognitive Science of Guangdong Province, South China Normal University, Guangzhou, China; 2 Department of Psychology, National University of Singapore, Singapore

## Abstract

Human risk-taking attitudes can be influenced by two logically equivalent but descriptively different frames, termed the framing effect. The classic hypothetical vignette-based task (Asian disease problem) and a recently developed reward-based gambling task have been widely used to assess individual differences in the framing effect. Previous studies treat framing bias as a stable trait that has genetic basis. However, these two paradigms differ in terms of task domain (loss vs. gain) and task context (vignette-based vs. reward-based) and the convergent validity of these measurements remains unknown. Here, we developed a vignette-based task and a gambling task in both gain and loss domains and tested correlations of the framing effect among these tasks in 159 young adults. Our results revealed no significant correlation between the vignette-based task in the loss domain and the gambling task in the gain domain, indicating low convergent validity. The current findings raise the question of how to measure the framing effect precisely, especially in individual difference studies using large samples and expensive neuroscience methods. Our results suggest that the framing effect is influenced by both task domain and task context and future research should be cautious about the operationalization of the framing effect.

The framing effect is a decision-making bias in which presenting the same option in different ways (e.g., positive vs. negative) can reverse people’s risk preference (i.e., risk aversion in the positive frame; risk seeking in the negative frame)^1^,^2^. After Kahneman and Tversky’s seminal paper introduced risky choice framing in 1981, the classic paradigm to examine the framing effect is using written vignettes that describe hypothetical scenarios and presents a choice between a sure and a risky outcome in positive and negative frames^1^,^2^,^3^,^4^. For example, in the well-known Asian disease problem, a majority of participants (72%) were risk averse when programs were positively framed based on a description of lives saved; however, preference reversal occurred when the same outcomes were negatively framed based on a description of lives lost (78%)^5^. Prospect theory accounts for this as a consequence of participants coding positive outcomes as gains and negative outcomes as losses, inducing risk averse and risk seeking preferences respectively^5^,^6^.

In recent years, another novel personal framing decision-making task introduced by De Martino *et al*.’s^7^ has been widely used in neuroimaging and genetic studies^7^,^8^,^9^,^10^,^11^,^12^. In this gambling task, participants were endowed with an initial amount (e.g., $50). They were then asked to choose between a sure amount and a gamble. The gamble was represented graphically using a pie chart that showed the chances of winning and losing as portions of the pie. The sure option was framed in one of two ways (e.g., keep $20 or lose $30 of the initial endowment). Although the two formulations are obviously equivalent, the sure option was chosen more often when it was framed positively (with the word ‘keep’) than when it was framed negatively (with the word ‘lose’) than the gamble option. This reward-based gambling task is similar to the vignette-based task in the construct.

To date, many studies have examined how individual differences in framing effect can be explained by psychological and biological traits. Specifically, some studies using different vignette-based tasks provide evidence that individual differences in personality are correlated with the framing effect^13^,^14^,^15^; for example, people who are high in need for cognition are less sensitive to the framing effect^16^,^17^. Similarly, the framing effect seen in risky task scenarios relates to various “big five” personality traits; for instance, people scoring low on openness have a tendency to show the risk preference reversal^18^. More importantly, several genetic studies using reward-based gambling task have found that genetic factors have a strong impact on the individual difference in susceptibility to framing. For example, individuals homozygous for the short (s) allele at the serotonin transporter gene-linked polymorphic region (5-HTTLPR) are more sensitive to framing compared with the long (la) group using a reward-based gambling task in the gain domain^7^,^9^,^11^. Recently, another genetic study found that the Met allele of COMT Val158Met polymorphism (rs4680) carriers show a greater framing effect than the Val/Val homozygotes as the former gambled more than the latter in the loss frame. Moreover, the gene-behavior association are mediated by resting-state functional connectivity between orbitofrontal cortex and bilateral amygdala^19^. Taken together, these studies indicate that there are stable individual traits that are associated with individual differences in susceptibility to framing effects and framing bias seems to be biologically determined. The underlying assumption therefore, is that individual susceptibility to framing manipulation is trait based and can be measured using different experimental tasks.

However, the convergent validity of these tasks has yet to be examined (e.g., reward-based gambling tasks in the gain domain and vignette-based tasks in the loss domain). Convergent validity is agreement between measures of the same construct assessed by different methods^20^,^21^. A successful evaluation of convergent validity shows that a test of a concept is highly correlated with other tests designed to measure the same concept. For instance, the scores a test of mathematics skills can be correlated with scores on other tests that are also designed to measure basic mathematical ability. High positive correlations between the test scores would be evidence of convergent validity. If measures from different methods are not correlated with each other, these methods may measure different traits rather than the same trait.

Notably, the two classic paradigms differ in task domains and task contexts (e.g., vignette-based task in the loss domain or gambling task in the gain domain), which may result in different framing effects. In the present study, domain meant the valence of the problem context (e.g., potential gain or potential loss in a final outcome), whereas frames represented how the advantage of the option (positive) or the disadvantage of the option (negative) was portrayed in the description outcome expectation^22^. Framing effects in hypothetical written vignettes are always observed in a loss problem domain (e.g., Asian disease problem, cancer problem and job loss problem)^23^, but research has not examined the variety of framing effects in a similar gain problem domain. Many studies on decision-making have shown that people weigh losses more heavily than equivalent gains and thus individuals’ risk-taking attitudes are diverse in the domain of gains and losses^24^,^25^,^26^. A recent study using a modified gambling task also found stable framing effects in the gain and loss domains (i.e., the total amount will be won or lost as a prerequisite in the context), but they appear to be associated with differential brain responses^22^. It is worth noting that a difference in risk attitude in different domains (i.e., risk aversion in a gain domain and risk seeking in a loss domain) is usually termed a reflection effect (i.e., whether the outcomes are gains or losses)^27^. Reflection effects involve real gains and losses, while framing effects involve the same choice outcomes as though they are gains or losses^27^,^28^,^29^. From this perspective, another question of interest in this study is whether an individual’s framing effect in a gain domain is correlated with the framing effect in a loss domain.

To address this question, we used multiple measures of framing to test their convergent validity. Specifically, a within-subjects design was used to examine the correlations among the framing effects across different task contexts and domains. That is, we conducted a modified reward-based gambling task^22^ and vignette-based task^2^ to test framing effects in the gain and loss domains, respectively. Importantly, the main purpose of this study was to investigate whether framing effects in two classic framing manipulations (i.e., hypothetical written vignettes-based task in the loss domain and real gambling task in the gain domain) were correlated. In addition, we tested the relationship between the framing effect in the gain domain and the loss domain, and the correlation between the framing effect in the gambling task and the vignette-based task. This allowed us to infer whether the framing effect was the same across contexts and domains or, alternatively, whether it was modulated by contexts and domains. Based on a vast literature that framing effect was correlated with certain personalities and genes, we predicted that the two classic paradigms designed to measure framing should be highly correlated with each other. On the other hand, since the two paradigms might involve different psychological processes, for example, Fagley and Miller (1997) demonstrated that several variables influence the effect of framing, including features of the decision problem such as problem contexts or who the affected people will be^30^, we predicted that the two types of tasks would not be correlated with each other.

## Materials and Methods

### Participants

One hundred and fifty-nine volunteers (80 male; mean age ± SD, 20.90 ± 1.88 years) participated in task 1 and task 2. A set of comprehension questions was presented to check if participants understood the game instructions. All participants answered the questions correctly. Participants received a base payment (¥25, about $4) and extra earnings from the gambling tasks, depending on their performance. The study was approved by the Ethics Committee of the School of Psychology at South China Normal University. The methods were carried out in accordance with the approved guidelines. Written, informed consent was obtained from each participant, and all participants were informed of their right to discontinue participation at any time. During the debriefing session, the majority of subjects were found to be unaware of any biasing effects that followed the experiment.

### Experimental paradigms

#### Reward-based gambling task

In task 1, we used a modified gambling task^22^ to examine how positive and negative frames influenced risk-taking behavior in both gain and loss domains. During an instruction period, participants were familiarized with the task and given ten practice trials.

**Figure 1:**
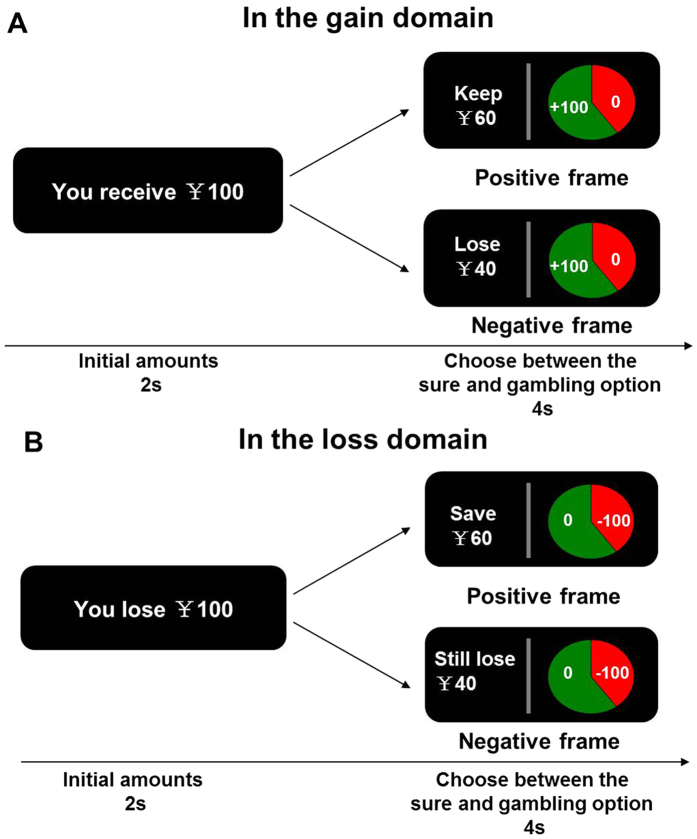
The gambling paradigm. At the beginning of each trial, a text message indicating the starting amount of money was presented for 2 s. The amount was a gain in the gain domain (**A**) and a loss in the loss domain (**B**). The participants were informed that they may or may not receive this initial amount of money, depending on their subsequent choices. Then a sure option was shown on the left of the screen and a gambling option was shown on the right of the screen for 4 s. The sure option was presented in a positive frame or negative frame in both domains, while a gambling option was presented as a pie chart depicting the frequency of winning (i.e., keep all of the initial reward in the gain domain or lose nothing in the loss domain) or losing (i.e., receive nothing in the gain domain or lose the initial amount in the loss domain). Participants were asked to make a decision within 4 s without feedback.

There were two different blocks, including a gain and loss block. Each block had a positive and negative frame. In the gain block (Fig. 1A), on each trial, participants were first shown a text message (for 2 s) indicating the starting amount of money that they would receive. Participants were informed that they would not be able to retain the whole of this initial amount, but would next have to choose between a sure and a gamble option presented in the context of two different frames. The sure amount was framed as the amount of the initial endowment participants would keep in the positive frame condition or as the amount of the initial endowment participants would lose in the negative frame. In the loss block (Fig. 1B), participants were first shown a message indicating the starting amount of money that they would lose. The sure amount was framed as the amount participants would save in the positive frame or as the amount participants would still lose in the negative frame. The gamble option was represented by a pie chart depicting the frequency of winning or losing in each trial. Participants in the gain domain block were told that they would gain all initial money if they won the gamble and would gain nothing if they lost the gamble. In the loss block, they would lose no money if they won the gamble and would lose the entire amount at stake if they lost the gamble. The expected values of the sure and gamble options were equivalent in each trial, and also mathematically equivalent between frames. The sure option was presented on the left side of the screen and the gamble option was presented on the right side of the screen. Participants were given 4 s to respond by pressing the left or right button. They were also told that during the task they would not receive feedback concerning the outcomes of their decisions. The inter-trial interval was 2 seconds.

Four different starting amounts were used in the task (¥25, ¥50, ¥75, and ¥100) and there were four different percentages of winning or losing in a given trial (20%, 40%, 60%, and 80%). Each combination of the starting amounts and the percentages was presented twice, resulting in a total of 64 game trials (32 positive frames, 32 negative frames) in each block. In two blocks, the starting amounts and the percentages of winning or losing were both fully balanced between the frame conditions. The gamble option was identical for both frames and represented by a pie chart depicting the frequency of winning or losing, and experimental conditions were randomized within the blocks. We also included 12 catch trials in each block to ensure that participants remained actively engaged in the decision-making task throughout the course of the experiment^7^. In these catch trials, in both frames, expected outcomes for the sure and gamble options were markedly unbalanced: in half of the trials (‘gamble weighted’) the gamble option was highly preferable (e.g., 95% probability of winning by taking the gamble option versus a sure choice of 50% of the initial amount) and for the other half of trials (‘sure weighted’) the sure option was preferable (e.g., 5% probability of winning by taking the gamble option versus a sure choice of 50% of the initial amount). As in the main experimental trials, the catch trials were also presented in either a positive or a negative frame. Participants were told that their performance in the task determined how much they would be awarded at the end of the task. One trial in the gain domain and one trial in the loss domain were randomly chosen and implemented. Unknown to the participants, if the final outcome was losing, participants received no reward. Thus, losing money meant winning no money in the end.

### Vignette-based task

In task 2, we used a modified vignette-based task^2^ to examine how positive and negative frames influenced risk-taking behavior in both gain and loss domains. Before the task, participants were familiarized with the task and given four practice trials.

**Figure 2:**
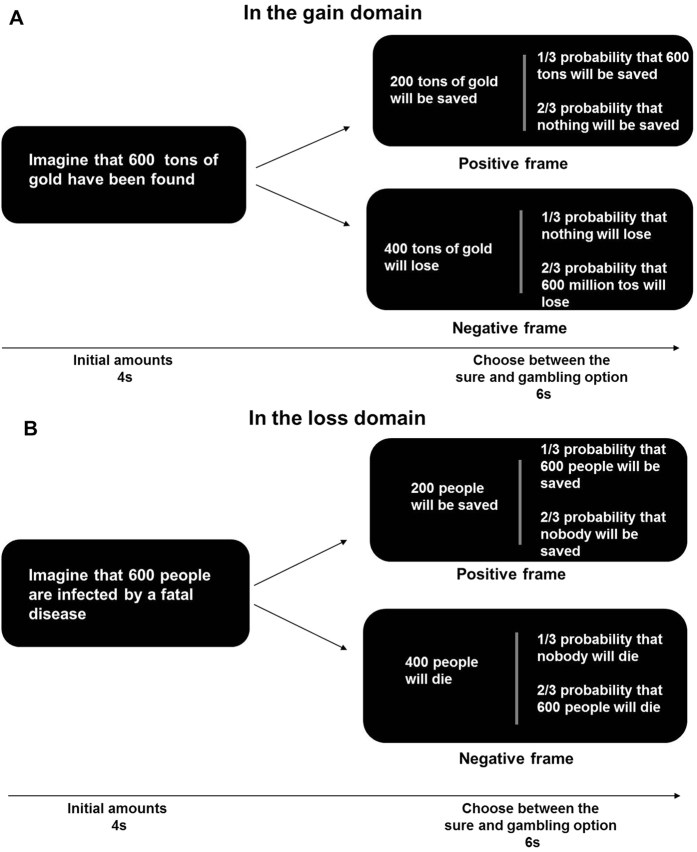
The vignette-based paradigm. At the beginning of each trial, the social problem with initial amount was shown for 4 s. The amount was a gain in the gain domain (**A**) and a loss in the loss domain (**B**). The participants were informed that the social problem with initial amount may or may not happen, depending on their subsequent choices. Then a sure option was shown on the left of the screen and a gambling option was shown on the right of the screen for 6 s. The sure option was presented in a positive frame or negative frame in both domains, while a gambling option was depicted as the frequency of a successful outcome (i.e., keep all of the initial amount in the gain domain or lose nothing in the loss domain with probability 1/3) or losing (i.e., save nothing in the gain domain or lose the initial amount in the loss domain with probability 2/3). Participants were asked to make a decision within 6 s without feedback.

At the beginning of each trial, the social problem with initial amounts was shown (for 4 s), and then participants had to choose between a sure and a gamble option presented in the context of two different frames within 6 s. There were 20 trials (16 game trials and 4 catch trials) in each block. In the gain block (Fig. 2A), a novel gold discovery task was used to investigate the framing effect in the gain domain. These were presented in one scenario and two different size contexts (i.e., ‘600/6 tons of gold have been found, but gold mining would mean suffering a certain amount of loss’). Half of the choice problems were framed positively in terms of the expected amount of gold saved while half of the problems were framed negatively in terms of the expected amount of gold lost. So 16 trials included 2 framing conditions × 1 scenario of gold discovery × 2 starting amounts × 4 repetitions. The probability of a successful outcome in the gamble option was 1/3. In the loss block (Fig. 2B), a modified life-death problem was used to investigate the framing effect in the loss domain. These modified life-death problems were presented in two scenarios: disease infection and terrorist kidnapping, with two different group sizes (e.g., ‘600/6 people have been kidnapped by terrorists and all hostages would die if the rescue were not carried out’). Half of the choice problems were framed positively in terms of the expected number of lives saved while half of the problems were framed negatively in terms of the expected number lives lost. Thus 16 trials included 2 framing conditions × 2 scenarios of the life-death problem × 2 starting amounts × 2 repetitions. The probability of the gamble options was constant as in the gain block (i.e., 1/3). In two blocks, the starting amounts and the probabilities of gaining or losing were both fully balanced between the framing conditions, and experimental conditions were randomized within the blocks.

We also included 4 catch trials in each block as in task 1. In these catch trials, in both the gain and loss frames, expected outcomes for the sure and gamble option were markedly unbalanced: half of the trials were ‘gamble weighted’ and the other half of trials were ‘sure weighted’. The sure option was presented on the left side of the screen and the gamble option was presented on the right side of the screen. Participants were told that during the task they would not receive feedback concerning the outcomes of their decisions. All blocks were presented in a fixed order (i.e., gambling task in the gain domain; vignette-based task in the loss domain; gambling task in the loss domain; vignette-based task in the gain domain).

### Data analysis

Eight participants’ data were removed from the analysis because their accuracy on catch trials in the two tasks was 3 standard deviations lower than the average, leaving a final sample of n = 151 (77 male) for analysis. The remaining subjects showed high accuracy on catch trials (mean ± SD > 0.85 ± 0.17), indicating their continued engagement with the task throughout the experiment. To examine the framing effect, two-way repeated measures ANOVAs using the domain (gain/loss) and the frame (positive/negative) as independent factors and the frequency of gambling choices as the dependent variable was performed to analyze the data from the gambling task and the vignette-based task separately. In these analyses, the Greenhouse–Geisser correction for non-sphericity was applied where appropriate. Additionally, each framing manipulation had a positive and negative frame that was presented in separate parts of the battery. Thus, using the frequency of choosing the gambling option in the negative frame minus the frequency of choosing the gambling option in the positive frame as an index of the framing effect, we further investigated the relationship among framing effects across different domains and different contexts.

## Results

### Framing effect in the gambling task

**Figure 3:**
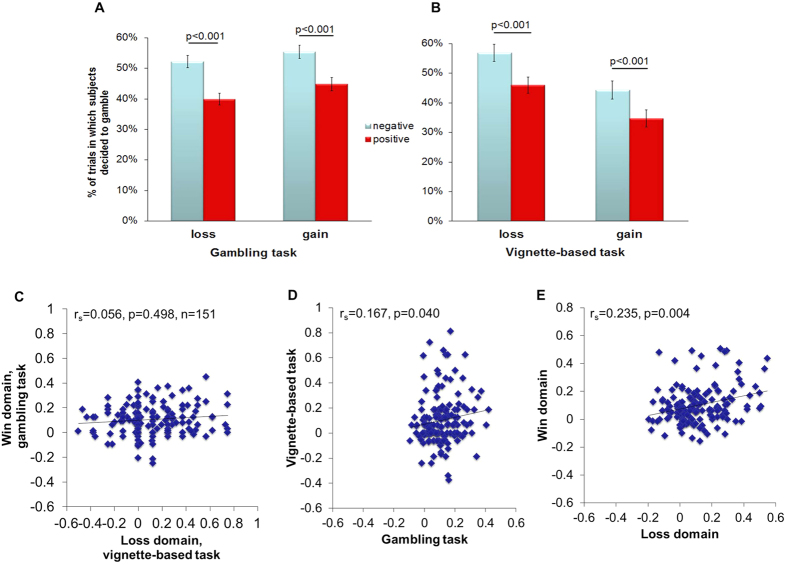
Behavioral results. In the gambling task (**A**) and vignette-based task (**B**), the frequency of gambling choices (mean, SE) was significantly higher in the negative frame than in the positive frame, regardless of domain. The framing effect in the gambling task in the loss domain showed no significant correlation with that in the vignette-based task in the gain domain (**C**). The framing effect in the gambling task and the framing effect in the vignette-based task were significantly correlated (**D**). The framing effect in the gain domain and the framing effect in the loss domain were significantly correlated (**E**).

In the gambling task, participants revealed a preference for gambling choices in the negative frame (53.8% ± 1.8%, mean ± SE) compared to the positive frame (42.3% ± 1.8%, F_(1,150)_ = 190.610, p < 0.001, η_p_^2^ = 0.560). There was also a significant difference between the loss (46% ± 1.9%) and gain domain (50.1% ± 2.1%, F_(1,150)_ = 4.932, p = 0.028, η_p_^2^ = 0.032). The interaction effect was not significant (F_(1,150)_ = 1.496, p = 0.223, η_p_^2^ = 0.010), suggesting no difference in the degree of the framing effect in the loss domain and the gain domain. However, paired sample t tests showed that the frequency of the gambling option in the negative frame (52.2% ± 2%) was significantly higher than that in the positive frame (39.9% ± 1.9%, t_152_ = 10.509, p < 0.001, *d* = 0.855; Fig. 3D) in the loss domain, and similarly, the frequency of the gambling option in the negative frame (55.5% ± 2.1%) was significantly higher than that in the positive frame (44.9% ± 2.1%, t_152_ = 10.611, p < 0.001, *d* = 0.864; Fig. 3A) in the gain domain, suggesting that framing effect is significant either in the gain domain or in the loss domain. Across different percentages (20%, 40%, 60%, 80%) and starting amounts (¥25, ¥50, ¥75, ¥100), the frequency of choosing the gambling option in the negative frame was significantly higher than that in the positive frame (p < 0.01), both in the gain domain and the loss domain.

### Framing effect in the vignette-based task

In the vignette-based task, we found behavior patterns similar to those seen in the gambling task. The frequency of gambling choices was significantly higher in the negative frame (50.6% ± 2.5%) than in the positive frame (40.4% ± 2.4%, F_(1,150)_ = 40.088, p < 0.001, η_p_^2^ = 0.211). There was a significant difference between the gain (39.5% ± 2.8%) and loss domain (51.5% ± 2.6%, F_(1,150)_ = 17.776, p < 0.001, η_p_^2^ = 0.106). The interaction effect was not significant (F_(1,150)_ = 0.287, p = 0.593, η_p_^2^ = 0.002), suggesting that the degree of the framing effect in the loss domain and the gain domain did not differ. However, paired sample t tests showed that the frequency of the gambling option in the negative frame (57% ± 2.9%) was significantly higher than that in the positive frame (46% ± 2.8%, t_152_ = 5.108, p < 0.001, *d* = 0.416; Fig. 3B) in the loss domain, and similarly, the frequency of the gambling option in the negative frame (44.3% ± 3%) was significantly higher than that in the positive frame (34.7% ± 2.9%, t_152_ = 4.921, p < 0.001, *d* = 0.405, Fig. 3E) in the gain domain, suggesting that framing effect is significant either in the gain domain or in the loss domain. Across different starting amounts (600/6), the frequency of choosing the gambling option in the negative frame was significantly higher than that in the positive frame (p < 0.001).

### Correlations between the two measurements

First, we found no significant correlation between the framing effect of the vignette-based task in the loss domain and the framing effect of the gambling task in the gain domain (Spearman correlation: r_s_ = 0.056, p = 0.498, n = 151; Fig. 3C). Second, there was a significant correlation between the framing effect of the gambling task and the vignette task (r_s_ = 0.167, p = 0.040, n = 151; Fig. 3D) when combining the gain and loss domains, and between the loss domain and the gain domain (r_s_ = 0.235, p = 0.004, n = 151; Fig. 3E) when combining the gambling and vignette tasks. Third, a significant correlation was found between the gain domain and the loss domain in the gambling task (Pearson correlation: r = 0.164, p = 0.045, n = 151) and in the vignette-based task (r_s_ = 0.178, p = 0.029, n = 151), respectively. But we observed no significant correlation between the gambling task and vignette-based task in the loss domain (r_s_ = 0.144, p = 0.078, n = 151) or gain domain (r_s_ = 0.027, p = 0.742, n = 151), respectively.

## Discussion

Across two different task contexts and domains, we demonstrate significant framing effects, suggesting that the framing effect can be robustly elicited in either vignette-based or gambling tasks in different domains. Importantly, we found no significant correlation between two commonly used measures–a vignette-based task in the loss domain and a gambling task in the gain domain–suggesting that framing effects may vary across task contexts and the valence of domains. Such low correlation between two classic measures could be used as evidence against their convergent validity. It raises the concern that whether these two classic paradigms both are valid methods to measure individual differences in framing effects during decision making or not.

Our correlation results lead us to conclude that an individual’s framing effect may be modulated by different task contexts and the valence of domains^13^,^31^. Although numerous studies have found that individual differences such as gender and thinking styles (e.g., rational and intuitive) are correlated with the framing effect^32^,^33^,^34^,^35^, there are still some divergent results, with no evidence that need for cognition and gender differences are predictors of risk attitudes^15^,^36^,^37^,^38^. One possibility is that task contexts and domains may modulate the magnitude of the framing effect. For example, participants were not paid in the vignette-based task and it looked much more abstract than the gambling task. Decision makers in personal-related tasks (e.g., reward-based gambling task) may need more consideration. Previous study has found that the requirement to think more about a choice is believed to prompt additional cognitive processing regarding the options^16^. Thus, cognitive psychologists, decision scientists, and economists should pay much attention to different framing measurements when discussing the framing effect on decisions.

There is now an increasing interest in the knowledge of two physical substrates responsible for behavior: the brain and the genome^39^,^40^. Recently, a meta-analysis of neuroimaging studies of risky choice framing effects has found that three brain regions are the key neural correlates of framing effects in life-death, monetary and other types of problems, such as the right inferior frontal gyrus (IFG), the left anterior cingulate cortex (ACC) and the left amygdala^41^. Intriguingly, these investigations also provided evidence that framing effect in two common measures (i.e., a vignette-based task and a gambling task) involves differential activation of separate neural substrates. While left amygdala and ACC played an important role in gambling tasks in the gain domain^7^,^8^,^9^,^10^, the right dorsolateral prefrontal cortex, parietal cortex and right IFG had influence on vignette-based tasks in the loss domain^2^,^42^. Although it is unclear whether these regions are specifically related with the framing effect in different tasks, the results seem to suggest that different psychological processes are engaged in different framing tasks.

In addition, as mentioned previously, existing research has shown that the genetic factors influence the framing effect^9^,^11^,^19^, which suggests that the framing effect for an individual is stable as a trait. But one limitation of these studies is the use of one task in one domain to test small samples of individuals’ risk attitudes, which may not provide enough evidence to conclude that individual differences can predict people’s framing effect. The low convergent validity of two classic framing measures is a potential limitation to the understanding of framing effect as a trait. Thus, the current findings argue that individual differences research using behavioral tasks should assess the convergent validity of different behavioral tasks. In this way, it may provide more evidence that people’s susceptibility to the framing of choice outcomes is a trait and has genetic basis, since convergent validity means the instrument is related to other measures of the same construct and related constructs^43^. Moreover, recent genetic study using a delayed discounting task found that oxytocin and estrogen receptor polymorphisms temper accelerated cellular aging in young females who are more inclined to make impatient choices. However, the authors admitted that it is unable to conclusively disentangle two potential underlying mechanisms of their findings, namely, impatience leads to the interaction effect between leukocyte telomere length and genetic sensitivity or, alternatively, such biological factors lead to impatience (state-dependent model)^44^. Thus, in order to effectively explain the relationship between the genes and individual susceptibility to a stable decision bias, further research is needed to test whether the genetic factor is associated with such decision bias under different tasks.

It is surprising that participants’ framing effects in the gain domain were accordant with those in the loss domain when combining two tasks. There was a similar positive correlation between the gain and loss domains in the gambling task and that in the vignette-based task as well. These findings may be in line with a previous study showing that what really matters in risk taking behavior is the different descriptions of outcome, not the valence of task domains^45^. The correlation of framing effects between the domain of gains and losses may further support the threat and opportunity hypothesis—that is, if decision makers are able to translate an objective threat (i.e., a description of loss in a loss problem domain) into an opportunity (i.e., an expectation of positive outcome), then it could be expected that they could translate an objective opportunity (i.e., a description of gain in a gain problem domain) into a threat (i.e., an expectation of negative outcome), because individuals could shift the reference point based on the valence of domains^46^. In addition, regardless of the valence of domains, the framing effect in the gambling task was correlated with the effect in the vignette-based task, suggesting that people’s risk-taking attitude in different tasks may develop in the same way. However, previous studies have provided evidence that decision makers motivated by incentives are more sensitive to framing effects^3^,^5^, such as in the gambling task. Notably, we found no correlation between the two tasks in the gain domain or between the two tasks in the loss domain, meaning that task context and domain may have an interactive effect on the magnitude of the framing effect. It is not clear how participants would encode the context and domain variables in the two different tasks. Kühberger *et al*.’s^29^ meta-analysis introduced reflection versus framing, as well as context, as factors. Inspection of their Table 3 is informative for the present paper and shows what also is found here: framing is more relevant to risk attitude than reflection, while the domains and contexts also have an influence, but mainly as nuisance variables^29^. In sum, our study suggests that individual differences in the framing effect are task-dependent (e.g., task contexts and domains).

There are several limitations that are worth mentioning. First, our research relies on within-subjects designs to test for framing effects. It is not clear what the results would have looked like in a more customary one-shot framing study. Using both positive and negative frames for each participant could promote hypothesis guessing of strategies or could lead to attenuation of framing effects. However, consistent with previous studies using repeated trials, we nevertheless found framing effects. Using repeated trials might allow researchers to have more reliable measures of the framing effect. Besides, most subjects were found to be unaware of any biasing effects that followed the experiment during the debriefing session. Second, one of our key findings is a lack of correlation between the vignette-based task in the loss domain and the gambling task in the gain domain. The two classic tasks have been used to measure individual differences in the framing effect. The underlying mechanisms explaining why the two measures were not correlated with each other remain unclear.

The current findings have a wide range of implications for our understanding of how a person’s framing effect might interact with the valence of domains and task context. If the framing effect is task-dependent, future studies should be cautious about drawing conclusions based on correlations involving individual differences in framing. In order to better examine the framing effect, future research should further examine the underlying mechanisms mediating the framing effect in gain and loss domains across different task contexts.

## Additional Information

**How to cite this article**: Zhen, S. and Yu, R. All framing effects are not created equal: Low convergent validity between two classic measurements of framing. *Sci. Rep.* **6**, 30071; doi: 10.1038/srep30071 (2016).

## References

[b1] Kühberger A. The influence of framing on risky decisions: A meta-analysis. Organ. Behav. Hum. Decis. Process. 75, 23–55 (1998). 9719656 10.1006/obhd.1998.2781

[b2] Zheng H., Wang X. & Zhu L. Framing effects: Behavioral dynamics and neural basis. Neuropsychologia 48, 3198–3204 (2010). 20600178 10.1016/j.neuropsychologia.2010.06.031

[b3] Levin I. P., Schneider S. L. & Gaeth G. J. All frames are not created equal: A typology and critical analysis of framing effects. Organ. Behav. Hum. Decis. Process. 76, 149–188 (1998). 9831520 10.1006/obhd.1998.2804

[b4] Strough J., Karns T. E. & Schlosnagle L. Decision‐making heuristics and biases across the life span. Ann. N. Y. Acad. Sci. 1235, 57–74 (2011). 22023568 10.1111/j.1749-6632.2011.06208.x PMC3755606

[b5] Tversky A. & Kahneman D. The framing of decisions and the psychology of choice. Science 211, 453–458 (1981). 7455683 10.1126/science.7455683

[b6] Kahneman D. & Tversky A. Prospect theory: An analysis of decision under risk. Econometrica: Journal of the Econometric Society 47, 263–291 (1979).

[b7] De Martino B., Kumaran D., Seymour B. & Dolan R. J. Frames, biases, and rational decision-making in the human brain. Science 313, 684–687 (2006). 16888142 10.1126/science.1128356 PMC2631940

[b8] Guitart-Masip M., Talmi D. & Dolan R. Conditioned associations and economic decision biases. Neuroimage 53, 206–214 (2010). 20600994 10.1016/j.neuroimage.2010.06.021 PMC2923756

[b9] Roiser J. P. . A genetically mediated bias in decision making driven by failure of amygdala control. J. Neurosci. 29, 5985–5991 (2009). 19420264 10.1523/JNEUROSCI.0407-09.2009 PMC2686091

[b10] Xu P. . Neural basis of emotional decision making in trait anxiety. J. Neurosci. 33, 18641–18653 (2013). 24259585 10.1523/JNEUROSCI.1253-13.2013 PMC3834062

[b11] Crişan L. G. . Genetic contributions of the serotonin transporter to social learning of fear and economic decision making. Social cognitive and affective neuroscience 4, 399–408 (2009). 19535614 10.1093/scan/nsp019 PMC2799947

[b12] Smith D. V., Sip K. E. & Delgado M. R. Functional connectivity with distinct neural networks tracks fluctuations in gain/loss framing susceptibility. Hum. Brain Mapp. 36, 2743–2755 (2015). 25858445 10.1002/hbm.22804 PMC4736507

[b13] Del Missier F., Mäntylä T. & Bruin W. B. Decision-making competence, executive functioning, and general cognitive abilities. J. Behav. Decis. Making 25, 331–351 (2012).

[b14] Toplak M. E., West R. F. & Stanovich K. E. Rational thinking and cognitive sophistication: Development, cognitive abilities, and thinking dispositions. Dev. Psychol. 50, 1037 (2014). 24188038 10.1037/a0034910

[b15] LeBoeuf R. A. & Shafir E. Deep thoughts and shallow frames: On the susceptibility to framing effects. J. Behav. Decis. Making 16, 77–92 (2003).

[b16] Simon A. F., Fagley N. S. & Halleran J. G. Decision framing: Moderating effects of individual differences and cognitive processing. J. Behav. Decis. Making 17, 77–93 (2004).

[b17] Smith S. M. & Levin I. P. Need for cognition and choice framing effects. J. Behav. Decis. Making 9, 283–290 (1996).

[b18] Levin I. P., Gaeth G. J., Schreiber J. & Lauriola M. A new look at framing effects: Distribution of effect sizes, individual differences, and independence of types of effects. Organ. Behav. Hum. Decis. Process. 88, 411–429 (2002).

[b19] Gao X. . COMT Val158Met polymorphism influences the susceptibility to framing in decision‐making: OFC‐amygdala functional connectivity as a mediator. Hum. Brain Mapp. 37, 1880–1892 (2016). 26917235 10.1002/hbm.23142 PMC6867526

[b20] Campbell D. T. & Fiske D. W. Convergent and discriminant validation by the multitrait-multimethod matrix. Psychol. Bull. 56, 81 (1959). 13634291

[b21] Guo B., Aveyard P., Fielding A. & Sutton S. Testing the convergent and discriminant validity of the Decisional Balance Scale of the Transtheoretical Model using the Multi-Trait Multi-Method approach. Psychology of Addictive Behaviors 22, 288 (2008). 18540726 10.1037/0893-164X.22.2.288

[b22] Yu R. & Zhang P. Neural evidence for description dependent reward processing in the framing effect. Front. Neurosci. 8, 1–11 (2014). 24733998 10.3389/fnins.2014.00056 PMC3973918

[b23] Schneider S. L. Framing and conflict: Aspiration level contingency, the status quo, and current theories of risky choice. J. Exp. Psychol. Learn. Mem. Cogn. 18, 1040 (1992). 1402709 10.1037//0278-7393.18.5.1040

[b24] Tom S. M., Fox C. R., Trepel C. & Poldrack R. A. The neural basis of loss aversion in decision-making under risk. Science 315, 515–518 (2007). 17255512 10.1126/science.1134239

[b25] Guo X. . Increased neural responses to unfairness in a loss context. Neuroimage 77, 246–253 (2013). 23562770 10.1016/j.neuroimage.2013.03.048

[b26] Zhou X. & Wu Y. Sharing losses and sharing gains: increased demand for fairness under adversity. J. Exp. Soc. Psychol. 47, 582–588 (2011).

[b27] Fagley N. S. A note concerning reflection effects versus framing effects. Psychol. Bull. 113, 451–452 (1993).

[b28] Wang X. T. Framing effects: Dynamics and task domains. Organ. Behav. Hum. Decis. Process. 68, 145–157 (1996). 8954876 10.1006/obhd.1996.0095

[b29] Kühberger A., Schulte-Mecklenbeck M. & Perner J. The effects of framing, reflection, probability, and payoff on risk preference in choice tasks. Organ. Behav. Hum. Decis. Process. 78, 204–231 (1999). 10343064 10.1006/obhd.1999.2830

[b30] Fagley N. S. & Miller P. M. Framing effects and arenas of choice: Your money or your life? Organ. Behav. Hum. Decis. Process. 71, 355–373 (1997).

[b31] Del Missier F., Mäntylä T. & Bruine de Bruin W. Executive functions in decision making: An individual differences approach. Thinking Reasoning 16, 69–97 (2010).

[b32] Shiloh S., Salton E. & Sharabi D. Individual differences in rational and intuitive thinking styles as predictors of heuristic responses and framing effects. Pers. Individ. Dif. 32, 415–429 (2002).

[b33] Charness G. & Gneezy U. Strong evidence for gender differences in risk taking. J. Econ. Behav. Organ. 83, 50–58 (2012).

[b34] Levin I. P., Snyder M. A. & Chapman D. P. The interaction of experiential and situational factors and gender in a simulated risky decision-making task. J. Psychol. 122, 173–181 (1988).

[b35] Byrnes J. P., Miller D. C. & Schafer W. D. Gender differences in risk taking: A meta-analysis. Psychol. Bull. 125, 367 (1999).

[b36] Schubert R., Brown M., Gysler M. & Brachinger H. W. Gender specific attitudes towards risk and ambiguity: An experimental investigation. Mimeo, Center for Economic Research, Swiss Federal Institute of Technology (Working Paper, 2000).

[b37] Levin I. P. & Chapman D. P. Risk taking, frame of reference, and characterization of victim groups in AIDS treatment decisions. J. Exp. Soc. Psychol. 26, 421–434 (1990).

[b38] Takemura K. The effect of decision frame and decision justification on risky choice. J. Psych. Res. 35, 36–40 (1993).

[b39] Robinson G. E., Grozinger C. M. & Whitfield C. W. Sociogenomics: Social life in molecular terms. Nat. Rev. Genet. 6, 257–270 (2005). 15761469 10.1038/nrg1575

[b40] Set E. . Dissociable contribution of prefrontal and striatal dopaminergic genes to learning in economic games. Proc. Natl. Acad. Sci. USA 111, 9615–9620 (2014). 24979760 10.1073/pnas.1316259111 PMC4084431

[b41] Wang X., Rao L.-L. & Zheng H. Neural Substrates of Framing Effects in Social Contexts: A Meta-Analytical Approach. Social Neuroscience (2016). 10.1080/17470919.2016.1165285 27007688

[b42] Gonzalez C., Dana J., Koshino H. & Just M. The framing effect and risky decisions: Examining cognitive functions with fMRI. J. Econ. Psychol. 26, 1–20 (2005).

[b43] Cohen R. J., Swerdlik M. E. & Sturman E. D. Psychological testing and assessment: An introduction to tests and measuremen 8^th^ edn (Mc Graw-Hill, 2012).

[b44] Yim O.-S. . Delay discounting, genetic sensitivity, and leukocyte telomere length. Proc. Natl. Acad. Sci. USA 113, 2780–2785 (2016). 26903639 10.1073/pnas.1514351113 PMC4790989

[b45] Mandel D. R. Gain-loss framing and choice: Separating outcome formulations from descriptor formulations. Organ. Behav. Hum. Decis. Process. 85, 56–76 (2001). 11341817 10.1006/obhd.2000.2932

[b46] Highhouse S. & Paese P. W. Problem domain and prospect frame: Choice under opportunity versus threat. Pers. Soc. Psychol. B. 22, 124–132 (1996).

